# Phase-synchronized 40 Hz tACS and iTBS effects on gamma oscillations

**DOI:** 10.1162/IMAG.a.140

**Published:** 2025-09-10

**Authors:** Benedikt Glinski, Mohammed Ali Salehinejad, Kuri Takahashi, Asif Jamil, Fatemeh Yavari, Min-Fang Kuo, Michael A. Nitsche

**Affiliations:** Department of Psychology and Neurosciences, Leibniz Research Centre for Working Environment and Human Factors, Dortmund, Germany; Department of Psychology, Ruhr-University Bochum, Bochum, Germany; School of Cognitive Sciences, Institute for Research in Fundamental Sciences (IPM), Tehran, Iran; Department of Child and Adolescent Psychiatry, Psychosomatics and Psychotherapy, Medical Faculty, RWTH Aachen University, Aachen, Germany; Division of Neuropsychiatry and Neuromodulation, Department of Psychiatry, Massachusetts General Hospital and Harvard Medical School, Boston, MA, United States; Bielefeld University, University Hospital OWL, Protestant Hospital of Bethel Foundation, University Clinic of Psychiatry and Psychotherapy, Bielefeld, Germany; German Centre for Mental Health (DZPG), Bochum, Germany

**Keywords:** gamma oscillations, memory, Alzheimer’s disease, transcranial alternating current stimulation, transcranial magnetic stimulation, theta burst stimulation, prefrontal cortex

## Abstract

Gamma oscillations play a crucial role in core cognitive functions such as memory processes. Enhancing gamma oscillatory activity, which is reduced in Alzheimer’s Disease, may have therapeutic potential, but effective interventions remain to be determined. Previous studies have shown that phase-synchronized electric and magnetic stimulation boosts brain oscillatory activities at theta, alpha, and delta frequency bands in different ways. The high-frequency gamma frequency band remains to be investigated. This study applies novel noninvasive brain stimulation techniques, namely phase-locked 40-Hz intermittent theta-burst stimulation (iTBS) and transcranial alternating current stimulation (tACS), and explores gamma oscillation changes in the brain. Thirty healthy young participants randomly underwent 40-Hz tACS (1), 40-Hz iTBS (2), two combined interventions (phase-locked iTBS to tACS peak sine wave or tACS trough sine wave) (3–4), and a sham condition (5). The target regions were the left and right dorsolateral prefrontal cortex and were stimulated by simultaneous tACS and iTBS. Gamma oscillatory activities (for 2 hours after intervention) were monitored following each intervention. Our results show that all stimulation protocols enhanced 40-Hz oscillatory power. The iTBS-tACS Peak shows the most significant and stable increase in gamma oscillatory activities (up to 2 hours), followed by 40-Hz tACS and 40-Hz iTBS. 40-Hz tACS and 40-Hz iTBS had the strongest acute effects (up to 30 minutes) on induced gamma oscillations, while 40-Hz tACS most consistently induced gamma oscillations for up to 2 hours in overall resting EEG data. Phase-synchronizing iTBS with tACS at 40 Hz and the very 40 Hz tACS alone targeting the dorsolateral prefrontal cortex may be a viable approach for inducing and stabilizing gamma oscillatory activity, particularly in conditions where endogenous gamma oscillations are attenuated, such as Alzheimer’s Disease.

## 1 Background

The prevalence of Alzheimer’s disease (AD) is expected to rise threefold by 2050, making AD a serious global health threat (Nandi et al., 2022). Its growing burden is reflected in rising costs, from 2.8 trillion USD in 2019 to a projected 16.9 trillion USD by 2050 (Nichols et al., 2022). Besides this current trend, treatment plans for patients mostly focus on symptom management (Pardo-Moreno et al., 2022). In addition, new therapeutics targeting amyloid beta (Aβ) plaques, a hallmark of AD, offer limited benefits and risk adverse effects like brain bleeding (Woloshin & Kesselheim, 2022). This underscores the need for innovative treatments. A recent treatment approach involves non-invasive brain stimulation (NIBS) that includes techniques like transcranial magnetic stimulation (TMS) and transcranial electrical stimulation (tES) that can modulate brain activity.

In TMS, the brain is stimulated by applying single or repetitive magnetic pulses at a specific frequency to the skull, which results in an electrical current flow in the underlying brain tissue that leads to changes in electrical and biochemical brain activity (Kobayashi & Pascual-Leone, 2003). In particular, the repeated application of TMS pulses (rTMS) is widely used in neurology, psychiatry, and basic neuroscience to induce long-term potentiation (LTP) and long-term depression (LTD) -like plasticity effects (Siebner & Rothwell, 2003) and induce short-lasting changes in oscillatory neural activity in the cerebral cortex (Fuggetta et al., 2008; Thut et al., 2011; Thut & Miniussi, 2009). Similar physiological effects are observed for tES interventions, mostly in transcranial direct current stimulation, which can induce LTP- and LTD-like plasticity comparable to rTMS (Ghanavati et al., 2025; Stagg & Nitsche, 2011; Wischnewski et al., 2019). In addition, electrical stimulation with an alternating current, transcranial alternating current stimulation (tACS), interacts with endogenous oscillatory activity by aligning ongoing oscillatory brain activity to the weak exogenous current produced by tACS (Wischnewski et al., 2023). This mechanism is called entrainment. The entrainment effects of tACS outlast the acute stimulation phase, leading to longer-lasting changes in oscillatory activity than the short-lasting modulatory effect on oscillations produced by rTMS (Kasten et al., 2016).

Recent evidence suggests that Aβ plaques disrupt plasticity mechanisms and are associated with abnormal oscillatory brain activity in patients compared with healthy controls (Di Lorenzo et al., 2016; Jafari et al., 2020). Both of these processes can be modulated using NIBS interventions, suggesting their potential as promising strategies for AD treatment. Gamma oscillations play a crucial role in working memory as well as the formation and retention of declarative memory (Düzel et al., 2010; Howard, 2003; Lundqvist et al., 2016). Previous investigations have shown decreased power of gamma oscillations in the electroencephalogram (EEG) of AD patients (Casula et al., 2022) and in intracranial recordings of animal models (Etter et al., 2019; Lee et al., 2014). Reduced gamma oscillations are prominently observed in the hippocampus, visual cortex, auditory cortex, and prefrontal cortex, including the dorsolateral prefrontal cortex (DLPFC) (Blanco‐Duque et al., 2024; Mably & Colgin, 2018; Ramlakhan et al., 2020). Specifically, reduced gamma oscillations in the hippocampus are associated with impaired GABAergic interneuron activity, and alleviating these gamma impairments has been shown to restore learning and memory deficits (Mably & Colgin, 2018). Additional AD animal studies have reported post-treatment increased Aβ clearance (Dhaynaut et al., 2022; Murdock et al., 2024). These findings suggest that mitigating gamma disturbances could represent a novel approach for addressing these AD-related disturbances in AD. In this line, modulating 40 Hz gamma oscillations enhances Aβ clearance and cognition in preclinical and clinical studies (Chen et al., 2025; Etter et al., 2019; Zhou et al., 2022), yet challenges remain, including variable patient responses, limited evidence of intervention efficacy, and NIBS protocol optimization (Benussi et al., 2021; Manippa et al., 2024).

NIBS techniques aiming to modulate or entrain 40 Hz oscillatory activity could be a promising approach for AD treatment, and protocol optimization may improve results. A recent research approach focusing on combining different NIBS techniques showed that the combination of rTMS applied to the peak of the alternating current produced by tACS led to a stronger and longer-lasting increase of mid-range oscillatory power (theta and alpha EEG bands) (Briley et al., 2024; Hosseinian, Yavari, Biagi, et al., 2021; Hosseinian, Yavari, Kuo, et al., 2021), while for the low-frequency band (i.e., delta) rTMS applied to the trough of the alternating current was most effective (Takahashi et al., 2024). To our knowledge, no study has investigated the effects of this combined stimulation approach for the gamma band.

The present study investigated the impact of five NIBS protocols that entrain 40 Hz oscillatory activity in the DLPFC of healthy adults. The DLPFC was targeted due to its critical role in working memory and global cognition via gamma oscillations, the disruption of these oscillations in DLPFC by Aβ plaques in Alzheimer’s disease, and its accessibility for NIBS, unlike the hippocampus, making it a feasible target for NIBS research (Benchenane et al., 2011; Koch et al., 2024; Kumar et al., 2017; Roux et al., 2012). We hypothesized that applying intermittent theta burst stimulation (iTBS), a high-frequency rTMS protocol, at the peak of the sinusoidal waveform generated by tACS would produce greater and more sustained increases in 40 Hz oscillatory activity compared to iTBS applied at the trough, tACS alone, iTBS alone, or sham stimulation (Hosseinian, Yavari, Biagi, et al., 2021; Hosseinian, Yavari, Kuo, et al., 2021). This hypothesis is supported by findings from non-human primate studies and computational models, which demonstrate that when oscillatory activity becomes synchronized, in higher frequency ranges neurons undergo stronger depolarization during the positive phase (peak) of the applied current, whereas a hyperpolarized state occurs during the negative phase (trough) (Johnson et al., 2020; Reato et al., 2013; Tran et al., 2022). For delta frequency ranges, however, the noise reduction by tACS-induced hyperpolarization at the trough phase is assumed to dominate, and thus, in this case, the rTMS stimulus gains efficacy, and the rTMS-induced oscillation is strengthened (Takahashi et al., 2024).

## 2 Methods

### 2.1 Participants

Thirty healthy subjects (20 females, mean age = 26.54, SD = 3.74) were recruited from the Technical University Dortmund, Ruhr-University Bochum, and the surrounding community. All subjects had normal or corrected-to-normal eye vision, were right-handed, non-smokers, and had no history of neurological or psychiatric disorders, including seizures or epilepsy, CNS medication intake, metal implants, and current pregnancy. To further verify the health status of the subjects, an examination was carried out by a medical doctor before the start of the experiment. Written informed consent was signed by all subjects. The study conformed with the latest version of the Declaration of Helsinki and was approved by the ethics committee of the Leibniz Research Centre for Working Environment and Human Factors (ID: IfADo-196). Participants were monetarily reimbursed and allowed to withdraw from the study at any time.

### 2.2 Intervention

**Fig. 1.**
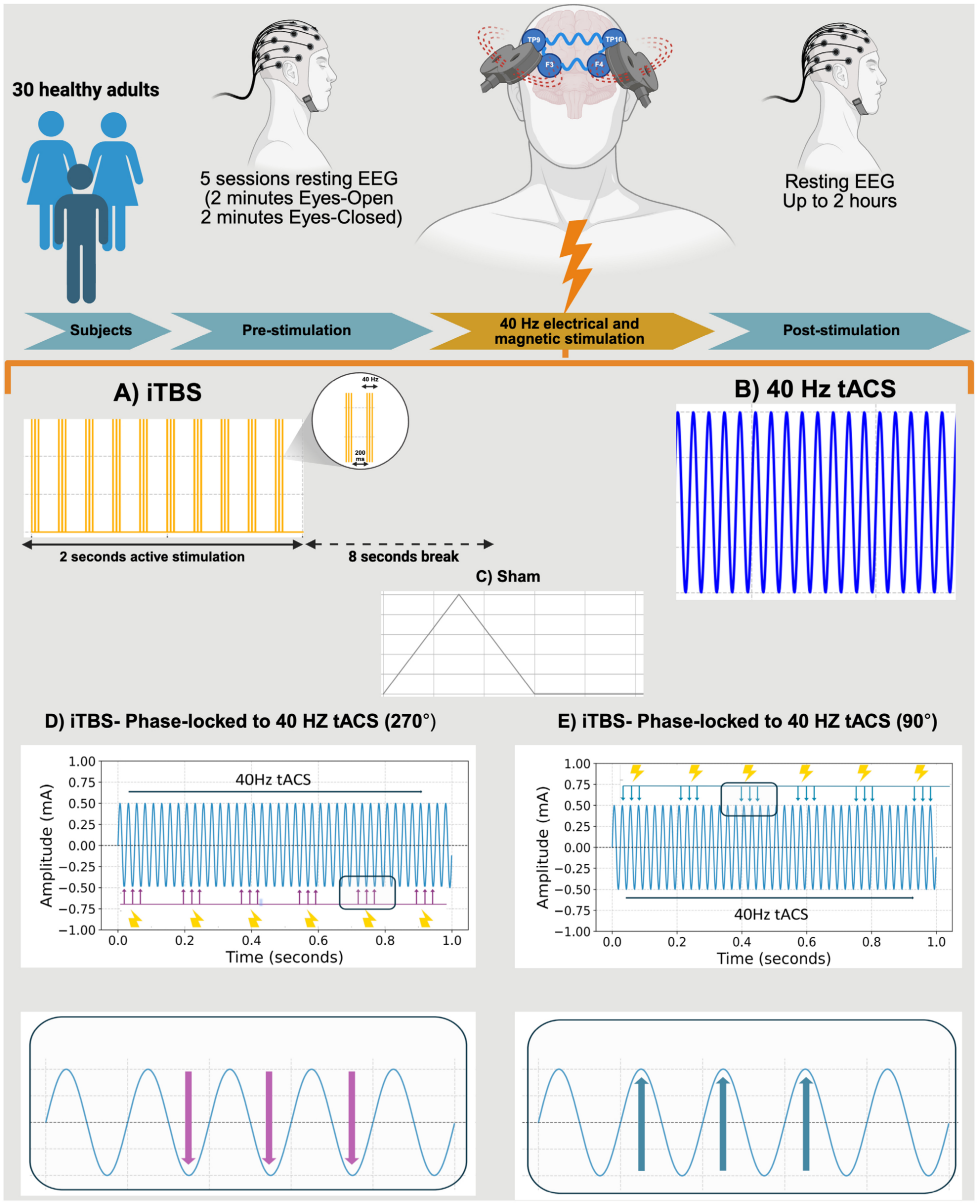
(A) Graphical representation of the study protocol. (B) Representation of the modified iTBS protocol. The protocol was applied four times in one experimental session. The iTBS cycles started at 0, 5, 10, and 15 minutes, leading to a total application of 2,400 pulses in one session per stimulation side. Active stimulation in the pattern seen in the representation was applied for 190 seconds per cycle (600 pulses). (C) Representation of 40 Hz tACS with an amplitude of 1 mA peak to peak. The current was delivered for 20 minutes per session. (D) Representation of the X-tACS Trough protocol. iTBS pulses were phase-locked to the trough (270 degrees) of the sine wave generated by tACS (represented by the arrows). The iTBS intervention was applied as described in B. (E) Representation of the X-tACS Peak protocol. iTBS pulses were phase-locked to the peak (90 degrees) of the sine wave generated by tACS (represented by the arrows). The iTBS intervention was applied as described in B.

Five interventions with different combinations of iTBS and tACS were tested: (1) *X-tACS Peak* protocol in which the applied iTBS pulses were synchronized to the peak of the target site’s tACS sinusoidal waveform (90° phase from wave onset), (2) *X-tACS Trough* protocol in which these iTBS pulses were synchronized to the trough of the tACS-generated sinusoidal waveform (270° phase from wave onset), (3) the intermittent 40 Hz theta burst protocol (iTBS alone) with sham tACS, (4) a 40 Hz tACS protocol (tACS alone) with sham iTBS, (5) and a sham protocol (see Fig. 1B–E). We used a modified iTBS protocol (intra-burst interval of 40 Hz instead of 50 Hz), applied at 80% of the individual subjects’ active motor threshold (AMT) four times within a 20-minute time frame starting at 0, 5, 10, and 15 minutes from the beginning of the intervention resulting in 2,400 delivered pulses for the whole intervention at each stimulation side. The tACS was delivered utilizing a Starstim 8-channel constant current, battery-powered electric stimulator (Neuroelectrics, Barcelona, Spain). Circular carbon rubber electrodes (2 cm radius, 12,57 cm^2^) were used for stimulation. During tACS, current was delivered with a 1 mA peak-to-peak amplitude with a 15-second ramp up at the beginning of the stimulation and a 15-second ramp down at the end of the stimulation. Sham tACS was applied by only ramping the current for 15 seconds up and 15 seconds down at the beginning and end of the stimulation respectively. A customized circuit was developed in-house to combine tACS and the modified iTBS protocol by phase-locking iTBS pulses to the tACS waveform. iTBS pulses were delivered using two figure-of-eight-shaped TMS coils. For sham iTBS, two different sham TMS coils (double 70 mm pCool-SHAM coil, Mag&More GmbH, Munich, Germany) generated audible clicks at the same intensity as an active coil without inducing an electromagnetic pulse. To minimize the delay between iTBS pulses from the two coils, the TMS devices were connected in series, with TTL signals used for triggering. An oscilloscope confirmed near-zero latency between the pulses. Details of the intervention are in the Supplementary Materials.

### 2.3 Neuronavigation

To control for movement of the TMS coils during the stimulation, an MR-based 3D-navigation system (LOCALITE GmbH, Germany) was utilized to monitor the orientation and position of the TMS coil to the 3D-head model and fiducials (nasion, left and right ear) of the participants derived from individual anatomical MRI scans. The T1-weighted structural scans were acquired by a Siemens 3T Prisma scanner based on the 3D-MP-RAGE sequence (TR = 2,600 ms, TE = 2.92 ms, flip angle = 12°, FoV read = 256 mm, FoVphase = 87.5%, 176 slices with 1 × 1 × 1 mm^3^ resolution).

### 2.4 Resting electroencephalography

EEG was recorded using a 64-channel EEG System (Bittium, NeurOne, Bittium Corporation, Finland) in a shielded room with stable light conditions. The Ag/AgCl EEG electrodes were attached to the head using high-viscosity electrolyte gel (SuperVisc, Easycap, Herrsching, Germany). All recordings were sampled with a sampling rate of 2,000 Hz. The online reference electrode was placed at FCz, and the ground electrode was placed at CPz. The EEG impedance was kept below 5 kOhms throughout the whole recording. Digital TTL triggers were transmitted to the EEG system by a server PC to indicate the start of a condition (eyes open or eyes closed) during the resting state recordings. For EEG preprocessing, BrainVision Analyzer version 2.2.2 (Brain Products GmbH, Gilching, Germany) was used. The pipeline was adapted from Salehinejad et al. (2021). EEG recordings were bandpass-filtered between 0.1 and 90 Hz (zero phase shift Butterworth filters) with a notch filter at 50 Hz to remove line noise. All electrodes were referenced to a common average reference montage. The vEOG signal from channel Fp2 was used for the correction of ocular artifacts using the Gratton and Coles method (Gratton et al., 1983) in line with our previous works (Salehinejad et al., 2021, 2022). Channels with artefactual signals were identified and spline-interpolated. Resting-state EEG recordings were then segmented into 2-second epochs; artifactual epochs containing EEG activity greater than a 100 mV peak-to-peak amplitude were automatically marked, visually inspected, and removed manually. Artifact-free segments were then transformed from the time to the frequency domain using Fast Fourier transform (Hanning window length 10%, zero-padded) and averaged.

### 2.5 Experimental design and procedure

Participants attended 5 experimental sessions (see Fig. 1A), all scheduled at the same time of the day (9 am or 1 pm) to control for arousal levels and sleep pressure (Salehinejad et al., 2021, 2022). Sessions were spaced at least 7 days apart to prevent carry-over effects. EEG recordings were conducted in a shielded, soundproof chamber with consistent lighting. Upon arrival, participants were seated in a reclined chair, and a 64-channel EEG cap was fitted according to the 10–10 international electrode placement convention. tACS electrodes were pre-placed over the target regions (F3, F4, TP9, and TP10). After the EEG setup, a vacuum pillow was used to stabilize the head position. The TMS intensity for iTBS was determined in each session separately to account for changes of cortical excitability. Baseline EEG recordings consisted of 2 minutes each of eyes open (EO) and eyes closed (EC) conditions. During the EO recording, participants fixated on a black cross against a white background at one meter eye distance.

### 2.6 Statistical analysis

Data were analyzed using R version 4.4.0 (R Core Team, 2024) and GraphPad Prism version 9.5.1 (GraphPad Software, Boston, MA, USA). Physiological data (EEG power spectrum and functional connectivity) were preprocessed and analyzed with Brainvision Analyzer, Fieldtrip, and Brainstorm toolbox (Oostenveld et al., 2011; Tadel et al., 2011). Figures were created using Prism 9.5.1, Microsoft PowerPoint, and MATLAB 2023b utilizing the Fieldtrip toolbox. Details of data analysis (physiology, behavior, blinding, and side effects) are presented in the Supplementary Materials.

## 3 Results

### 3.1 Data overview, blinding, and side effects

No significant differences were observed in tingling, burning, pain, or skin redness across stimulation conditions. However, a significant effect of stimulation on visual phenomena was detected (Supplementary Table S3). Post hoc analyses revealed significantly larger visual phenomena for tACS (t = 3.67, p = 0.001), X-tACS Trough (t = 3.54, p = 0.001), and X-tACS Peak (t = 3.61, p = 0.001), while iTBS again showed no significant difference (p = 0.801). Follow-up correlational analyses indicated a significant positive correlation (p = 0.015) with moderate strength (r = 0.45; see Supplementary Fig. S2) between visual phenomena and post-intervention 40 Hz oscillatory power in the frontal ROI only in the eyes open condition (EEG sensors: F3, F4, Fz, F1, F2, AF3, AF4, FC3, FC4, F5, F6) specifically for the tACS protocol in the EO condition. This correlation, however, was not significant at the whole-brain level (r = 0.35, p = 0.065). The remaining non-significant correlations between visual phenomena and induced 40 Hz gamma oscillations were as follows: r = 0.09 and r = 0.20 for the X-tACS Peak at the frontal ROI and whole-brain level, respectively, and r = -0.92 and r = 0.07 for the X-tACS Trough.

### 3.2 Stimulation effects on 40 Hz oscillatory power

The linear mixed model (LMM) analysis revealed no significant baseline differences between the absolute 40 Hz power of the different stimulation protocols in the EO, EC, and overall conditions in the frontal ROI and at the whole-brain level (Supplementary Fig. S3). Temporal dynamics of the interventions focusing on the frontal region of interest were analyzed using separate LMMs for the EO and EC recording conditions. The analyses revealed significant main effects of stimulation (EO: F_4,6979_ = 43.90, p < 0.001; EC: F_4,7111_ = 17.25, p < 0.001; overall: F_4,7076.7_ = 16.90, p < 0.001), time (EO: F_4,6978_ = 85.41, p < 0.001; EC: F_4,7111_ = 121.82, p < 0.001; overall: F_4,7076.2_ = 97.49, p < 0.001), and electrode (EO: F_10,6978_ = 5.36, p < 0.001; EC: F_10,7111_ = 4.01, p < 0.001; overall: F_10,7076.1_ = 8.01, p < 0.001), and a significant stimulation × time interaction (EO: F_16,6978_ = 5.86, p < 0.001; EC F_16,7111_ = 2.03, p < 0.001; overall: F_16,7076.1_ = 2.54, p < 0.001) (Table 1). Subsequent pairwise comparisons revealed temporal and stimulation-specific effects.

**Table 1.** LMM results: Significant main and interaction effects of stimulation, time and electrode on 40 Hz oscillatory power in the frontal ROI.

Condition	Factor	d.f.	F value	p value	ηp2
Eyes-open	stimulation	4, 6979	43.90	**<0.001**	0.02
	time	4, 6978	85.41	**<0.001**	0.06
	electrode	10, 6978	5.36	**<0.001**	0.00
	stimulation × time	16, 6978	5.86	**<0.001**	0.01
	stimulation × electrode	40, 6978	1.02	0.43	0.00
	electrode × time	40, 6978	0.65	0.96	0.00
	stimulation × time × electrode	160, 6978	0.38	1.00	0.00
Eyes-closed	stimulation	4, 7111	17.25	**<0.001**	0.00
	time	4, 7111	121.82	**<0.001**	0.06
	electrode	10, 7111	4.01	**<0.001**	0.00
	stimulation × time	16, 7111	2.04	**<0.01**	0.00
	stimulation × electrode	40, 7111	2.81	**<0.001**	0.02
	electrode × time	40, 7111	0.83	0.77	0.00
	stimulation × time × electrode	160, 7111	0.41	1.00	0.00
Overall (Eyes-open + Eyes-closed)	stimulation	4, 7076	16.89	**<0.001**	0.00
	time	4, 7076	97.49	**<0.001**	0.05
	electrode	10, 7076	8.01	**<0.001**	0.01
	stimulation × time	16, 7076	2.54	**<0.001**	0.00
	stimulation × electrode	40, 7076	1.97	**<0.001**	0.01
	electrode × time	40, 7076	0.86	0.72	0.00
	stimulation × time × electrode	160, 7076	0.39	1.00	0.00

Bold values show significant p values.

#### 3.2.1 40 Hz oscillatory power at frontal ROI

##### 3.2.1.1 Eyes open condition

Compared to baseline, X-tACS Peak, tACS, and iTBS significantly increased 40 Hz power during the first 30 minutes post-intervention (all p < 0.001), with no effect for X-tACS Trough and sham conditions. At the 30–60 minutes time window, all active protocols increased 40 Hz power (all p < 0.001) and remained significant until 120 minutes post-intervention (all p < 0.001). Sham stimulation did not produce significant changes besides the 90–120 minutes time window when an increased 40 Hz power was observed (t = 3.08; p = 0.010; Supplementary Table S8). When compared to the sham condition, X-tACS Peak, X-tACS Trough, tACS, and iTBS protocols significantly increased 40 Hz oscillatory power during the first 30-minutes post-intervention (all p < 0.05). The effects of X-tACS Peak, tACS, and iTBS stimulation were not statistically different from each other. Furthermore, X-tACS Peak, tACS, and iTBS stimulation protocols had a stronger increase of 40 Hz gamma oscillations compared to the X-tACS Trough protocol (all p < 0.05).

**Fig. 2.**
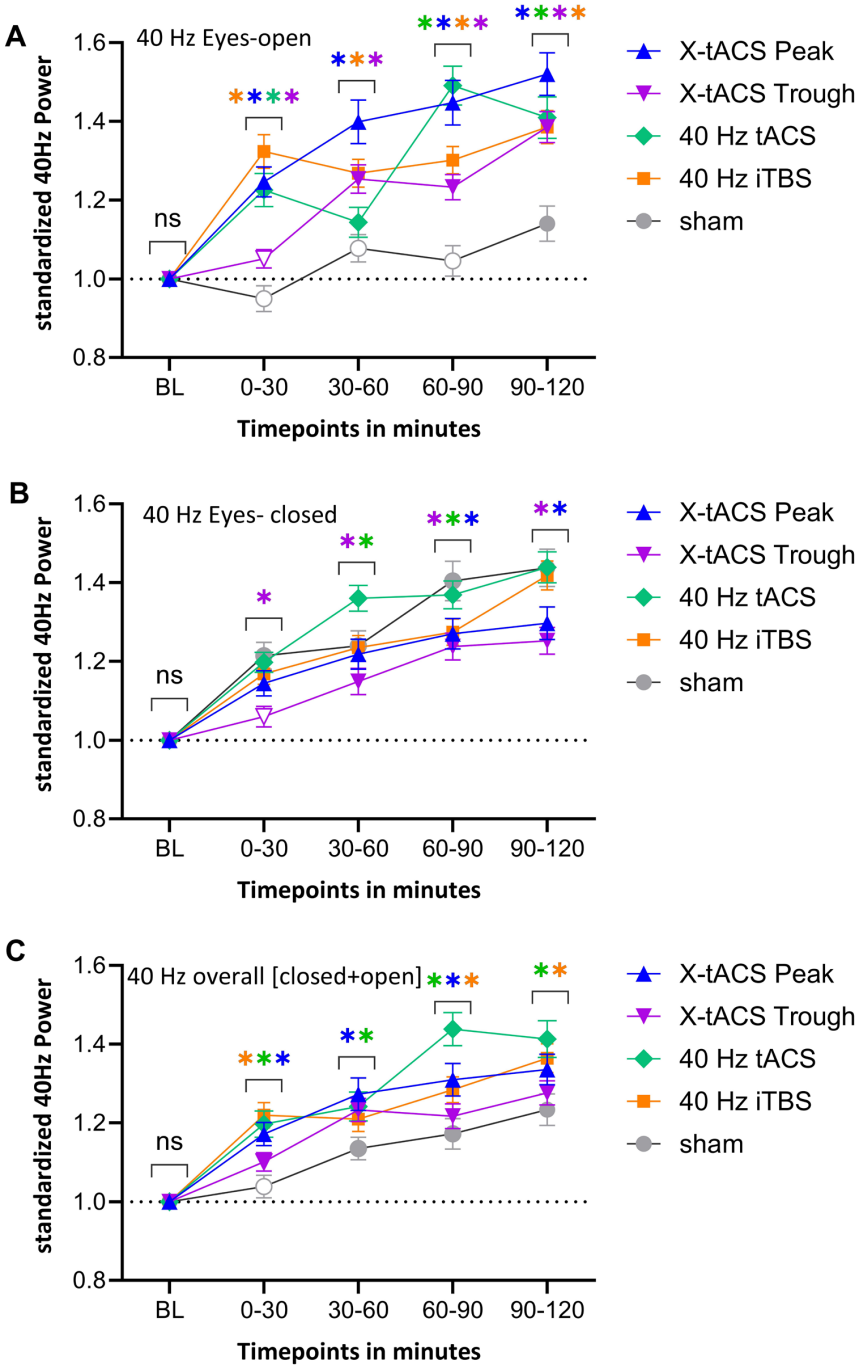
(A) Relative time-dependent changes in 40 Hz power for all stimulation protocols in the frontal ROI, compared to the baseline during **eyes-open** recordings, are shown. (B) Relative time-dependent changes of 40 Hz power in the frontal ROI are shown for all stimulation protocols in relation to the baseline for the **eyes closed** recordings. (C) Relative time-dependent changes in 40 Hz power for all stimulation protocols in the frontal ROI, compared to the baseline during **eyes-open+eyes closed (overall)** recordings, are shown. In a, b, and c, filled symbols indicate a significant difference compared to the baseline. Asterisks above the graph indicate a significant difference compared to sham (p < 0.05, FDR-corrected). Error bars represent the standard error of the mean.

In the 30–60 minutes time window, all protocols except tACS resulted in a significant increase in 40 Hz oscillatory power compared to the sham condition (all p < 0.01). Here, the X-tACS Peak protocol produced the most substantial enhancement, significantly surpassing the effects produced by X-tACS Trough, tACS, and iTBS (all p < 0.05). No other significant effects were found. In the 60–90 minutes time window, all protocols significantly increased 40 Hz oscillatory power compared to sham (all p < 0.001). X-tACS Peak and tACS showed the largest effects, both outperforming iTBS and X-tACS Trough (p < 0.001). Finally, during the 90–120 minutes time window, all protocols maintained significantly higher 40 Hz oscillatory power compared to sham (all p < 0.001). The X-tACS Peak stimulation condition induced a significantly larger increase than both X-tACS Trough and iTBS (all p < 0.05), but not compared to tACS (t = -1.99, p = 0.07). Differences between iTBS, X-tACS Trough, and tACS conditions were not statistically significant (Fig. 2A). For detailed statistics, see Supplementary Tables S7 and S8; for effects in other frequency bands, see Supplementary Figure S4.

##### 3.2.1.2 Eyes closed condition

Compared to the baseline, all interventions, including sham, except for X-tACS Trough during the first 30 minutes post-stimulation (t = 1.41; p = 0.18), significantly increased 40 Hz oscillatory power (all p < 0.01). When compared to the sham condition, X-tACS Trough resulted in decreased 40 Hz power during the first 30 minutes (t = -3.76, p < 0.01). No other significant differences were found compared to the sham condition. Compared to all other active protocols, X-tACS Trough significantly decreased 40 Hz oscillatory power (all p < 0.05). In the 30–60 minutes time, tACS increased 40 Hz power (t = 2.57, p = 0.03) whereas X-tACS Trough decreased 40 Hz power (t = -2.41, p = 0.03) when compared to sham. The increasing effect of tACS on 40 Hz power in the frontal region was higher than for all other tested protocols (all p < 0.05). No other significant effects were observed during this time window. At the 60–90 minutes post-intervention, all protocols except tACS reduced 40 Hz power compared to sham (all p < 0.001). The effect of tACS did not differ significantly from sham (t = -1.21; p = 0.31) but was significantly different from the X-tACS Trough protocol (t = 3.08; p < 0.01), which caused the greatest reduction. No other significant differences were observed between the protocols. Finally, during the period of 90–120 minutes, only the combined protocols significantly reduced 40 Hz power compared to sham (p < 0.05). This decreasing effect of X-tACS Peak and X-tACS Trough was also significant when compared to iTBS and tACS (p < 0.05). No significant difference was observed between X-tACS Peak and X-tACS Trough conditions (Fig. 2B; Supplementary Tables S9–S10; for effects in other frequency bands see Supplementary Fig. S4).

##### 3.2.1.3 Eyes closed + eyes open condition

Compared to baseline, all active stimulation protocols significantly increased 40 Hz gamma oscillation at all time points. The sham condition showed similar increases except during the first 30 minutes. When compared to the sham, in the first 30 minutes post-stimulation, 40 iTBS and tACS, followed by X-tACS Peak, significantly increased 40 Hz gamma oscillations compared to the sham. At 60–90 minutes post-intervention, these same protocols significantly enhanced 40 Hz gamma oscillations. Only tACS and X-tACS Peak enhanced 40 Hz gamma oscillations at 30–60 minutes post-intervention. At 90–120 minutes post-intervention, only 40 iTBS and tACS increased 40 Hz gamma oscillations. 40 Hz tACS was the only stimulation protocol that significantly increased gamma oscillation in all time points.

### 3.3 40 Hz oscillatory power at the whole-brain level

The effects of stimulation on the whole-brain level were investigated using separate LMMs for EO and EC recording conditions. Significant main effects were found for stimulation (EO: F_4,38638_ = 236.19, p < 0.001; EC: F_4,38309_ = 149.64, p < 0.001), time (EO: F_4,38637_ = 470.71, p < 0.001; EC: F_4,38309_ = 737.70, p < 0.001), electrode (EO: F_60,38637_ = 8.94, p < 0.001; EC: F_60,38309_ = 7.01, p < 0.001), and significant interactions for stimulation × time (EO: F_4,38637_ = 29.10, p < 0.001; EC: F_4,38309_ = 17.01, p < 0.001) and electrode × stimulation (EO: F_240,38637_ = 2.19, p < 0.001; EC: F_240,38309_ = 2.22, p < 0.001). No other significant effects were found (Table 2).

**Table 2.** LMM results: Significant main and interaction effects of stimulation, time, and electrode on 40 Hz oscillatory power on the whole-brain level.

Condition	Factor	d.f.	F value	p value	ηp2
Eyes-open	stimulation	4, 38638	236.19	**<0.001**	0.03
	time	4, 38637	470.71	**<0.001**	0.05
	electrode	60, 38637	8.94	**<0.001**	0.01
	stimulation × time	16, 38637	29.10	**<0.001**	0.01
	stimulation × electrode	240, 38637	2.19	**<0.001**	0.02
	electrode × time	240, 38637	1.06	0.24	0.00
	stimulation × time × electrode	960, 38637	0.50	1.00	0.01
Eyes-closed	stimulation	4, 38309	149.64	**<0.001**	0.02
	time	4, 38308	735.70	**<0.001**	0.07
	electrode	60, 38308	7.01	**<0.001**	0.01
	stimulation × time	16, 38308	17.01	**<0.01**	0.00
	stimulation × electrode	240, 38308	2.22	**<0.001**	0.01
	electrode × time	240, 38308	1.10	0.19	0.00
	stimulation × time × electrode	960, 38308	0.55	1.00	0.01

Bold values show significant p values.

#### 3.3.1 Eyes open condition

**Fig. 3.**
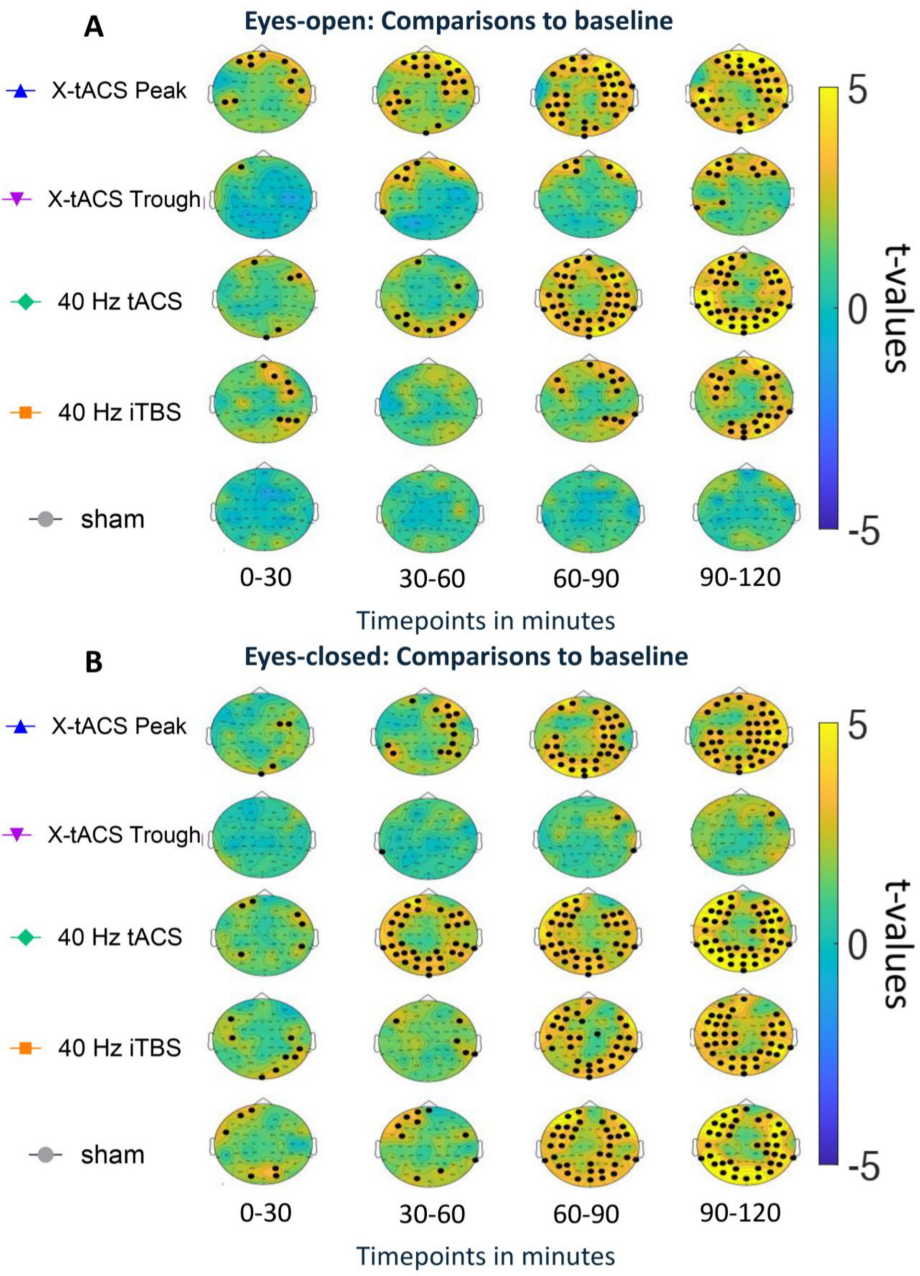
(A) Whole-brain changes in 40 Hz oscillatory power are shown at four-time windows following the intervention (0–30, 30–60, 60–90, and 90–120 minutes) relative to baseline during **eyes-open** recordings. T-values from post hoc tests are shown on topological graphs. Positive t-values indicate an increase in oscillatory power, while negative values indicate a decrease. Black dots indicate a significant difference from baseline (p < 0.05, FDR-corrected). (B) Whole-brain changes in 40 Hz oscillatory power across different time windows (0–30, 30–60, 60–90, and 90–120 minutes) are compared to the baseline for **eyes-closed** recordings. The t-values from post hoc tests are plotted on topographical graphs, with positive t-values indicating an increase and negative values indicating a decrease in oscillatory power. Black dots represent significant differences from the baseline (p < 0.05, FDR-corrected).

Compared to baseline, X-tACS Peak significantly increased 40 Hz oscillatory power in frontal and temporal regions of both hemispheres within 0–30 minutes. tACS enhanced 40 Hz power in frontal, parietal, and occipital regions, while iTBS increased it in the right frontal and parietal cortices. X-tACS Trough and sham stimulation showed no significant effects. From 30 to 60 minutes, both X-tACS Peak and Trough significantly enhanced 40 Hz oscillations, especially in frontal and temporal regions. tACS continued to significantly increase target frequency power, particularly in frontal and occipital regions, while iTBS and sham showed no significant changes. During the 60- to 90-minute interval, X-tACS Peak significantly increased 40 Hz power across frontal, temporal, parietal, and occipital cortices. X-tACS Trough’s earlier effects persisted, and tACS showed intensified 40 Hz power increases in the same regions. iTBS significantly increased 40 Hz power around stimulation sites, extending to the right parietal cortex. Sham stimulation showed no significant effects. In the 90- to 120-minute post-intervention, the significant effects of X-tACS Peak, X-tACS Trough, and tACS were maintained. X-tACS Trough also showed increased 40 Hz power in the left parietal cortex. iTBS effects intensified, with new significant increases in the occipital cortex, while sham stimulation remained ineffective (Fig. 3A).

**Fig. 4.**
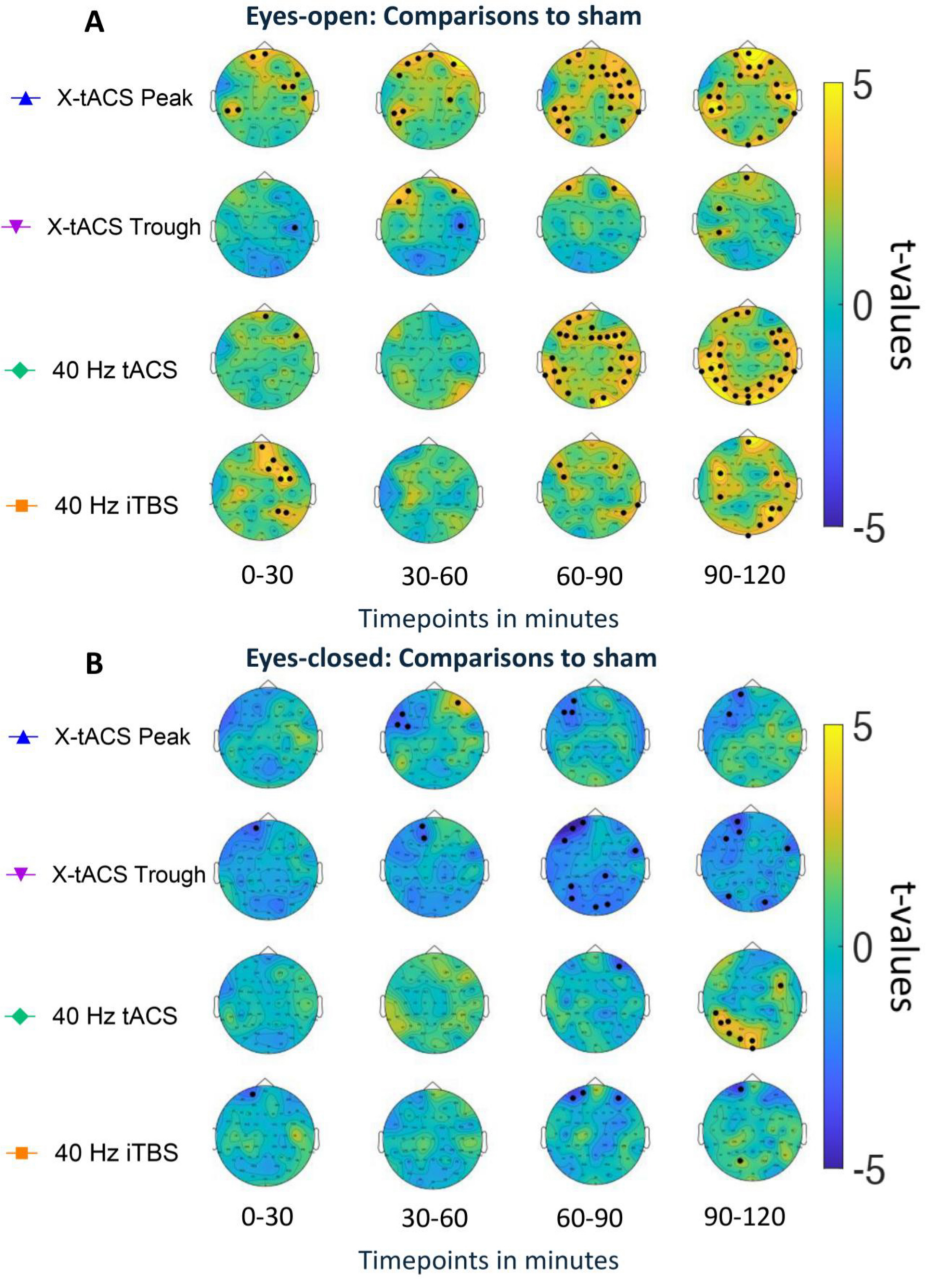
(A) Whole-brain changes of 40 Hz oscillatory power for the different time windows (0–30, 30–60, 60–90, and 90–120 minutes) are presented compared to the sham stimulation for the **eyes open** recordings. t-values obtained from the post hoc tests are plotted onto the topological graphs. Positive t-values indicate an increase in oscillatory power, whereas negative values indicate a decrease in oscillatory power. Black dots indicate a significant difference compared to the sham condition (p < 0.05, FDR-corrected). (B) Whole-brain changes of 40 Hz oscillatory power for the different time windows (0–30, 30–60, 60–90, and 90–120 minutes) are presented compared to the sham stimulation for the **eyes closed** recordings. t-values obtained from the post hoc tests are plotted onto the topological graphs. Positive t-values indicate an increase in oscillatory power, whereas negative t-values indicate a decrease in oscillatory power. Black dots indicate a significant difference compared to the sham condition (p < 0.05, FDR-corrected).

Compared to sham, X-tACS Peak increased 40 Hz oscillatory power in frontal and temporal cortices of both hemispheres within 0–30 minutes, while iTBS affected frontal and parietal cortices. Specifically, tACS increased 40 Hz power only in two frontal electrodes, and X-tACS Trough decreased oscillations in one right parietal electrode. The X-tACS Peak effects persisted from 30 to 60 minutes, and X-tACS Trough significantly increased 40 Hz power in the frontal cortex; tACS and iTBS showed no significant effects. From 60 to 90 minutes, X-tACS Peak’s effects intensified, with further increases in bihemispheric parietal and occipital cortices. X-tACS Trough’s effects continued, and tACS also significantly increased 40 Hz power across frontal, temporal, parietal, and occipital cortices. iTBS showed a focal increase around stimulation sites and in the right parietal cortex. During the 90- to 120-minute window, all active stimulation protocols maintained and intensified their effects, with X-tACS Trough also significantly increasing 40 Hz power in the left parietal cortex (Fig. 4A).

#### 3.3.2 Eyes closed condition

The X-tACS Peak significantly increased 40 Hz power in the right frontal and occipital cortices within the first 30-minute post-intervention, while tACS had similar effects in bilateral frontal and parietal cortices. iTBS increased 40 Hz power in the left frontal and right parieto-occipital cortices. Sham stimulation increased 40 Hz power in the left frontal and occipital regions, while X-tACS Trough showed no effect. Between 30 and 60 minutes, X-tACS Peak significantly enhanced 40 Hz power in the right frontal, temporal, and bilateral parietal cortices, and tACS increased it across bilateral frontal, temporal, parietal, and occipital cortices. iTBS moderately increased 40 Hz power in the frontal and right parietal regions. Sham stimulation also raised 40 Hz power in the bilateral frontal, parietal, and occipital cortices; X-tACS Trough had no effect. From 60 to 90 minutes, the effects of X-tACS Peak and tACS persisted and intensified, particularly in the temporoparietal and occipital cortices for X-tACS Peak. iTBS showed stronger effects on 40 Hz power in the frontal, temporal, parietal, and occipital regions, as did sham stimulation. X-tACS Trough still showed no effect. These effects persisted and intensified from 90 to 120 minutes (Fig. 3B).

There were no significant effects of stimulation protocols compared to the sham condition in the first 30-minute post-intervention. In the 30- to 60-minute window, both the X-tACS Peak and X-tACS Trough protocols showed a significant decrease in 40 Hz power in the left frontal region, with no other significant effects detected. This decrease persisted in the 60- to 90-minute period for both protocols, alongside significant decreases in the right temporal, left parietal, and occipital regions. The iTBS protocol also led to a significant decrease in frontal regions, with no other significant effects found. In the final 90- to 120-minute window, previous effects persisted, and a significant increase in 40 Hz power was observed in the left parieto-occipital region for the tACS protocol, with no other significant effects noted (Fig. 4B).

## 4 Discussion

This study investigated the impact of five NIBS protocols, developed via a combination of tACS and iTBS, on inducing and stabilizing 40 Hz gamma oscillations in healthy individuals. Specifically, we investigated the impact of 40-Hz tACS, 40-Hz iTBS, phase-locked 40 Hz iTBS to 40 Hz tACS peak sine wave, phase-locked 40 Hz iTBS to 40 HZ tACS trough sine wave, and a sham iTBS-tACS over the left and right dorsolateral prefrontal cortex. In addition to examining the local effects of the stimulation in the frontal lobe, we assessed the global impact of NIBS on 40 Hz gamma power across the entire brain. In what follows, we discuss major physiological findings.

### 4.1 40 Hz entrainment under NIBS interventions and induced gamma oscillations

Our major finding is that the combined protocol (40 Hz iTBS phase-locked to tACS) most effectively induced gamma activity in the EO recording, followed by 40 Hz tACS and 40 Hz iTBS alone. In the EC condition, iTBS protocols significantly decreased 40 Hz power compared to sham, while tACS alone most effectively boosted 40 Hz power between 30 and 90 minutes. The gamma-enhancing effects of tACS applied at the peak of oscillations partly replicate previous findings showing enhanced alpha and theta power in the EO condition using the same intervention (Hosseinian, Yavari, Biagi, et al., 2021; Hosseinian, Yavari, Kuo, et al., 2021), although the opposite pattern was observed for the delta band with tACS applied at the trough (Takahashi et al., 2024).

A possible explanation for the primary finding is that iTBS applied at the sine wave may have induced in-phase 40 Hz neuronal activity, facilitating tACS entrainment. Previous research has demonstrated that repetitive rTMS can transiently alter oscillations based on pulse frequency, causing a “phase reset” (Fuggetta et al., 2008; Lakatos et al., 2019) followed by short-lasting synchronization at the driving frequency (Thut et al., 2011). Thus, iTBS-induced 40 Hz activity may enhance alignment between endogenous and external oscillations, strengthening tACS entrainment. Additionally, some studies emphasize the importance of ongoing oscillatory activity during tACS, which iTBS can modulate. Another study (Kemmerer et al., 2022) showed that tACS at the individual alpha peak significantly modulated oscillatory power in that frequency, whereas +/- 2 Hz or sham stimulation was less effective, highlighting tACS’s frequency specificity. Similarly, frontoparietal tACS aligned with the individual theta peak, compared to non-individualized 4 Hz tACS or sham, led to stronger oscillatory power and network synchrony (Zhang et al., 2022). Consistent with this, the strongest modulation occurred at the 40 Hz target frequency, confirming tACS’s capacity to selectively entrain endogenous oscillations in a frequency-dependent manner. While oscillatory effects were also present in other bands like theta and alpha (Supplementary Fig. S4), they were less pronounced.

Another explanation concerns increased efficacy of iTBS to induce action potentials at the cortical level when the TMS pulses are applied to the peak of the tACS sine wave. TACS aligns ongoing natural oscillatory activity with its exogenous current (Wischnewski et al., 2023). Non-human primate studies, along with computational models, show that as oscillatory activity becomes aligned, the underlying neurons exhibit stronger depolarization during the positive phase (peak) of the applied current, while a hyperpolarized state is observed during the negative phase (trough) of the current (Johnson et al., 2020; Reato et al., 2013; Tran et al., 2022). Applying iTBS at the peak, when the neuronal membrane is depolarized and closer to the firing threshold, may facilitate action potential generation. Conversely, iTBS at the trough, during hyperpolarization, would likely be less effective. Consistent with this, Wischnewski et al. (2022) showed that cortical excitability depends on the phase of ongoing oscillations, with TMS pulses delivered during the peak or falling phase of the beta rhythm resulting in increased MEP amplitudes. This highlights the enhanced efficacy of TMS pulses during the depolarized phase and explains the stronger effects observed with the X-tACS Peak protocol.

Although the most pronounced increase in gamma oscillatory power occurred in the iTBS phase-locked to the peak of the tACS wave during the eyes eyes-open state, the specific mechanisms driving the differential effects of these interventional protocols require further investigation. A difference of the results of this study compared to our previous works (Hosseinian, Yavari, Biagi, et al., 2021; Hosseinian, Yavari, Kuo, et al., 2021) is that the stimulation effects did not transfer to the EC recording condition, while the same effect was seen for both EO and EC conditions in those studies. One possible reason for the greater intervention effects in the EO condition in the present study is the stronger high-frequency brain activity, including gamma rhythms, present when the eyes are open, compared to the general shift to lower frequencies, such as alpha rhythms, when the eyes are closed (Barry et al., 2007). The stronger effects observed in the EO condition may be due to the higher frequency oscillatory activity during the baseline state at the time of stimulation, which occurred during rest with the eyes open. Prior research indicates that baseline oscillatory activity at the target frequency is critical for the entrainment effects of tACS (Wischnewski et al., 2022). Similarly, in rTMS, the baseline brain state during stimulation influences oscillatory changes (Silvanto & Pascual-Leone, 2008), which may explain the enhanced effects in the EO condition.

### 4.2 Gamma oscillations under other interventions and brain states

The results of resting-state EEG analyses specifically during the eyes closed condition and overall (eyes open + eyes closed) strongly show that the 40 Hz tACS alone induced 40 Hz gamma power most effectivity and consistently up to 2 hours post-intervention. This may be because the eyes-closed state provides an arousal baseline, while the eyes-open condition provides an activation baseline (Barry et al., 2007). The superior effectiveness of 40 Hz tACS alone in inducing gamma power during eyes closed indicates the ability of tACS to linearly increase gamma power over time, when the brain is not further activated by visual input. This suggests that tACS might be the most effective protocol when brain activity and endogenous power oscillations are lower (Neuling et al., 2013). Conversely, increased arousal and visual engagement in the eyes-open state may amplify the effectiveness of all interventions, diminishing the relative superiority of tACS alone seen in the eyes-closed condition. The similar effects of tACS and the Peak protocol within the first 30 minutes of the eyes-open condition, with iTBS showing the largest effect, further suggest that the activation baseline allows multiple interventions to effectively modulate gamma power. The strong acute effect of 40 Hz iTBS and tACS alone (in the first 30 minutes) is thus another relevant observation.

### 4.3 Limitations, future directions, and therapeutic potential

The present study has several limitations. Firstly, it served as a proof-of-concept, examining the effects of the new stimulation protocols on oscillatory aftereffects and safety in a healthy population. Although no adverse events were reported, further research is needed to explore the stimulation effects across different healthy age groups and in clinical populations, such as those with mild cognitive impairment or Alzheimer’s disease, to enhance therapeutic outcomes and explore whether clinical translation is feasible or not. Additionally, we cannot discount the possibility that visual phenomena from the tACS intervention influenced the observed 40 Hz oscillatory changes in the frontal region of interest. Future studies should address these visual effects, especially with higher stimulation intensities, which may exacerbate them. Moreover, effective blinding techniques should be implemented to reduce the confounding effects of phosphenes during tACS application. Lastly, although our sample size aligns with previous studies and power analyses, increasing the sample size in future research is recommended to enhance statistical power, particularly in studies involving clinical populations.

The primary objective of the study was to evaluate the potential of various 40 Hz entraining NIBS interventions in inducing gamma oscillations, which are typically reduced in AD. While future studies should explore this in actual AD patients, the current findings are discussed in terms of clinical efficacy and feasibility below. The combined 40 Hz iTBS-tACS (phase-locked at the peak of the tACS wave) and 40 Hz tACS alone demonstrated particularly strong and long-lasting induced gamma oscillations, making them promising for therapeutic applications in AD. Regarding aftereffects, these protocols sustained gamma oscillations for up to 2 hours and exhibited linearly increasing effects during eyes-open conditions (combined protocol) and eyes-closed conditions (tACS alone). Nonetheless, a strong acute effect of the iTBS-alone intervention on gamma oscillations was also observed (during eyes-open and overall resting EEG states). However, the lack of prolonged aftereffects suggests that iTBS alone may not be as effective as the combined 40 Hz iTBS-tACS or 40 Hz tACS alone. Regarding feasibility, the combined protocols require a complex setup involving two TMS machines, one tES device, and a neuronavigator. While this setup may be practical in a laboratory setting, it might not be feasible or affordable for some clinics. Consequently, the use of 40 Hz tACS alone, which induced gamma oscillations for up to 2 hours, appears to be a more practical option. Alternatively, a modified approach to the combined protocol, such as stimulating only one hemisphere, should be explored in future studies to assess its comparative efficacy.

### 4.4 Conclusion

In conclusion, all protocols increased 40 Hz gamma oscillations during eyes-open recordings. The most effective protocol was the X-tACS Peak protocol, where the iTBS pulses were phase-locked to the peak of the tACS-generated sine wave. This finding is in accordance with findings from previous research with similar stimulation approaches. During eyes closed and for the overall state, the 40 Hz tACS condition alone was the most effective and consistent protocol in inducing gamma oscillation. Future studies should focus on the underlying mechanisms of the aftereffects produced by the stimulation protocols and implications for the transfer to behavioral modulations.

## Data and Code Availability

The data that supports the findings will be available in Zenodo with DOI:10.5281/zenodo.16088592 following a 1-year embargo from the date of publication.

## Author Contributions

Benedikt Glinski: Data curation, Formal analysis, Investigation, Methodology, Writing—original draft, and Visualization. Mohammed Ali Salehinejad: Data curation, Visualization, Formal analysis, Writing—original draft, Methodology, and Writing—review & editing. Kuri Takahashi: Data curation, Writing—review & editing. Asif Jamil: Methodology, Writing—review & editing. Fatemeh Yavari: Methodology, Writing—review & editing. Min-Fang Kuo: Conceptualization, Methodology, Supervision, Validation, Resources, Funding acquisition, and Writing—review & editing. Michael A. Nitsche: Conceptualization, Supervision, Methodology, Resources, Funding acquisition, and Writing—review & editing.

## Funding

This project has received funding from the Horizon 2020 research and innovation program of the European Union under grant agreement No 101017716 (Neurotwin).

## Consent Statement

All subjects provided written informed consent.

## Supplementary Material

Supplementary Material
